# A case for adoption of continuous albendazole treatment regimen for human echinococcal infections

**DOI:** 10.1371/journal.pntd.0008566

**Published:** 2020-09-17

**Authors:** Francesca Tamarozzi, John Horton, Marin Muhtarov, Michael Ramharter, Mar Siles-Lucas, Beate Gruener, Dominique A. Vuitton, Solange Bresson-Hadni, Tommaso Manciulli, Enrico Brunetti

**Affiliations:** 1 Istituto di Ricovero e Cura a Carattere Scientifico (IRCCS) Sacro Cuore Don Calabria Hospital, Department of Infectious-Tropical Diseases and Microbiology, Negrar, Verona, Italy; 2 Tropical Projects, Hitchin, United Kingdom; 3 Multi-Profile Hospital for Active Treatment “Kardzhali”, Gastroenterology Ward, Khardzhali, Bulgaria; 4 Department of Tropical Medicine, Bernhard Nocht Institute for Tropical Medicine, Department of Medicine, University Medical Center Hamburg-Eppendorf, Hamburg, Germany; 5 Instituto de Recursos Naturales y Agrobiología de Salamanca (IRNASA), Consejo Superior de Investigaciones Científicas (CSIC), Parasitology Group, Salamanca, Spain; 6 Division of Infectious Diseases, Department of Internal Medicine III, University Hospital Ulm, Ulm, Germany; 7 French National Reference Center for Echinococcosis, Besançon University Hospital, University Bourgogne Franche-Comté, Besançon, France; 8 Hepato-Gastroenterology and Tropical Medicine Units, University Hospital, Geneva, Switzerland; 9 Istituto di Ricovero e Cura a Carattere Scientifico (IRCCS) San Matteo Hospital Fundation–Unit of Infectious and Tropical Diseases, Pavia, Italy; 10 Department of Clinical, Surgical, Diagnostic and Pediatric Sciences, University of Pavia, Pavia, Italy; Universidad Peruana Cayetano Heredia, PERU

## Abstract

Cystic (CE) and alveolar (AE) echinococcosis are chronic, neglected parasitic diseases burdened by high morbidity and, for AE, by high mortality, if left untreated. CE and AE have a widespread distribution, including Europe. Albendazole (ABZ), a broad-spectrum benzimidazole drug widely used to treat parasitic infections, is the drug of choice for the management of CE and AE, and is parasitostatic on echinococcal metacestodes. In Europe, ABZ is licensed for interrupted “cyclic” treatment, for a maximum of 3 cycles. However, better efficacy with no increased side effects has been shown when the drug is administered continuously and for longer periods. Current international recommendations, on the basis of clinical, pharmacological, and biological studies, recommend continuous administration of ABZ for months to years for the treatment of CE and AE, and this schedule has been widely in use for the past 20 years. However, in Europe this internationally recommended schedule, with the exception of France, is technically “off-label”, and, as such, requires an informed consent by the patient and, in some countries, even precludes the reimbursement of the drug cost. Adding to the very high cost of the drug, frequent “out-of-stock” situation, and packaging format impractical for long therapies, these conditions put patients with CE and AE regularly at risk of treatment discontinuation and disease progression. European regulations envisage variations to marketing authorization, but postauthorization studies should be carried out by the holder of the license of the drug, in the form of randomized controlled trials. While such studies do not seem feasible and would probably not be ethically justified for CE and AE, European regulations envisage other possibilities in particular situations, which apply to CE and AE, but there is limited interest to invest in this perspective. We urge a coordination between stakeholders to find effective and feasible ways to take action to revise the benzimidazole dosage regimens for CE and AE and to ensure a fair, regular, and easy access to the appropriate treatment to those suffering from these serious diseases.

Albendazole (ABZ) and mebendazole (MBZ) are broad-spectrum anthelmintic drugs, included in the World Health Organization (WHO) list of essential medicines, and are used in the treatment of several, mainly metazoan, parasitic infections [1]. While most of these require single dose or relatively short treatment courses at low doses (e.g., soil transmitted helminthiasis or filariasis), cystic echinococcosis (CE) and alveolar echinococcosis (AE), the main echinococcal infections [2], are exceptions requiring long-term and high-dose treatment regimens [3, 4].

CE and AE are chronic, difficult to manage diseases, burdened by high morbidity and, for AE, by high mortality, if left untreated [5, 6]. They are caused by infection with the larval stage (metacestode) of the parasites *Echinococcus granulosus sensu lato* causing CE and *E*. *multilocularis* causing AE. In humans, *Echinococcus* larvae grow as cysts (CE) or lesions composed of an aggregate of microcysts (AE) in the liver and other organs after ingestion of parasite eggs from the environment [7]. Central Asian areas have the highest prevalence of both diseases; in Europe, CE is endemic in Mediterranean and Eastern countries, while AE occurs in Western-Central, Baltic, and Eastern countries [8]. A median of over 180,000 disability-adjusted life years (DALYs) have been estimated to be lost worldwide due to CE and over 680,000 DALYs for AE, with 95% confidence intervals reaching over 1 million DALYs for each disease [6]. The latter seem the most plausible figures due to the well-known problems of diagnosis and underreporting of these infections [9].

Antiparasitic drug treatment with benzimidazoles for CE and AE has been available for over 40 years, firstly using MBZ in the 1970s and then the more bioavailable and efficacious ABZ in the 1980s. Currently, ABZ is still the drug of choice for the management of CE and AE, and no alternative drugs are available [10, 11]. For AE, ABZ administration is necessary for all active infections [10]. For CE, the WHO Informal Working Group on Echinococcosis (WHO-IWGE) recommends ABZ administration as the sole treatment for uncomplicated abdominal CE in selected cyst stages, for disseminated infection, and as peri-interventional prophylaxis of secondary echinococcosis when surgical or percutaneous treatments are performed [10].

In Europe, ABZ is licensed at national level for interrupted “cyclic” treatment, consisting of 28 days of treatment followed by 14 days of interruption, generally for a maximum of 3 cycles [12]. In some countries (e.g., Portugal, Romania, and Spain), it is envisaged to extend the number of cycles for AE, or (e.g., Spain) treatment may be extended only for bone and cerebral localizations in case of CE. Only the French National Agency for Medicine and Health Products Safety (ANSM) accepted the opinion of national experts regarding continuous administration, and the drug registration document (May 24, 2005, and updated April 2, 2019) mentions that “the treatment is usually administered as 28-days courses, with an interruption of 14 days between courses. However, there is no evidence that a discontinuous treatment is mandatory, especially regarding drug tolerance, compared with continuous treatment. Therefore, a continuous administration without interruption between 2 courses can be done. It may even be recommended in disseminated clinical forms of the diseases, and in immunosuppressed patients” [Author’s translation] [13]. Since the original approvals of ABZ for CE and AE, continuous treatment with longer duration has been increasingly used by the medical community, based on a growing amount of data on safety and efficacy and over 30-year practical experience, and the WHO-IWGE Expert Consensus document since 1996 clearly recommends continuous treatment for both CE and AE [10, 14].

For AE, WHO-IWGE recommends ABZ treatment for a minimum of 2 years, if complete surgical resection of lesions is performed, and lifelong in virtually all other circumstances [10]. Continuous treatment was first used in difficult to control, advanced and disseminated cases; it was then extended to all cases because a clear improvement in the control of AE lesions progression was observed when cyclic treatment was switched to continuous intake [15, 16]. For CE, the optimal ABZ treatment schedule has not been fully defined yet, but a treatment duration between 3 to 6 months is generally prescribed [10, 17]. Horton [18] carried out a review of published and unpublished data collected over 12 years from the introduction of ABZ, administered with interrupted “cyclic” schemes. Granted that a meta-analysis was not carried out and that no cyst stages distribution was taken into account, an overall efficacy (cure and improved) of 71% was reported at the cyst level. Over the same period, Franchi and colleagues [19], using continuous 3- to 6-month ABZ administration, reported that, at the end of treatment, 82% of cysts had degenerative changes. More recently, Salinas and colleagues [20] used an interrupted treatment schedule on 64 active and transitional cysts, reporting a long-term success of 34%, while Larrieu and colleagues [21] used a continuous schedule and reported a long-term success of 96%. Despite the impossibility of rigorously comparing these studies due to the differences (and nonhomogeneous description) of the populations, cyst characteristics, and overall length of therapy, these results suggest a better efficacy of the continuous administration for the treatment of CE. When ABZ was registered for the treatment of echinococcosis, the interrupted “cyclic” regimen was chosen as a precaution because only limited data on the long-term toxicity were available. From published literature, direct comparison of adverse events rates occurring with the use of the 2 regimens is not possible. However, evidence gathered with the use of continuous administration, even when used for a very long time, show that common side effects, such as increase in liver enzymes or much rarer ones such as alopecia or leucopoenia, occur no more frequently and follow the same pattern when the drug is administered continuously or intermittently, in both AE and CE [16, 18, 19, 22]. In general, all reference centers in Europe (as well as in China [23] and in Latin America [21]) have abandoned the “cyclic” administration of ABZ for CE and AE.

Benzimidazole drugs in *Echinococcus* spp. metacestodes prevent cytoskeleton assembly by targeting parasite beta-tubulin [24]. Even after complete solidification is observed on imaging as the result of ABZ treatment, some CE cysts may reactivate after discontinuation of treatment [25, 26], indicating that ABZ did not kill all *Echinococcus* germinative cells [27]. This could be due to the limited affinity of ABZ for the beta-tubulin isoform most expressed in the germinative cells [27]. Thus, ABZ at the doses used in clinical practice is parasitostatic rather than parasitocidal, and this observation provides the biological rationale for the use of continuous as opposed to interrupted “cyclic” administration. To put it in the words of Teggi and colleagues [28], “intermittent administration could be more useful to the parasite than to the host,” a conclusion they reached just on the basis of clinical observation. These pharmacological and biological studies were conducted using *E*. *multilocularis*, as it is easier to use in vitro compared to *E*. *granulosus*; however, almost identical forms of beta-tubulin are also present in *E*. *granulosus* [27], supporting the hypothesis that the same mechanism might be involved in the limited efficacy of ABZ and reactivations after treatment interruption in both AE and CE.

All evidence strongly supports the use of continuous treatment with ABZ over the licensed interrupted “cyclic” regimen, if necessary for months to years in some cases [15, 29]. However, as highlighted recently by Horton [30], in Europe, this internationally recommended schedule, with the notable exception of France, is technically “off-label”, and, as such, requires an informed consent by the patient and, in some countries (such as Bulgaria), even precludes the reimbursement of the drug cost [31]. The French situation regarding ABZ licensing, which envisages continuous administration, seems to be related to the later date of registration, which allowed taking into account cumulative evidence and data from more recent publications. European regulations state that “it may be necessary in specific situations to complement the data available at the time of authorization with additional information concerning the efficacy of a medicinal product to address concerns that could not be resolved prior to the granting of the marketing authorization. […] a significant change may occur in the standard of care for the diagnosis, treatment or prevention of a disease, leading to the need to re-open discussions on the established benefit-risk balance of the medicinal product. […] if an improved understanding of the disease or the pharmacology of a medicinal product has brought into question the criteria used to establish the efficacy of the medicinal product at the time of the marketing authorization was granted, additional studies may be considered.” [32]. Among variations to marketing authorization are included “variation or addition of a new dosage,” and the circumstances that justify postauthorization studies include those when “the benefits of a medicinal product demonstrated in clinical trials are significantly affected by the use of the medicinal product under real-life conditions” [33]. Such postauthorization studies should be carried out by the holder of the license of the drug, in the form of randomized controlled trials [33]. However, while randomized controlled trials would be the ideal study design to address interrupted versus continuous ABZ administration and optimize treatment duration, such trials do not seem feasible. The major obstacles to the implementation of prospective randomized clinical trials to compare treatment arms in the field of echinococcosis are (1) the relative rarity of the condition, (2) the many different factors influencing clinical decision-making in each patient and treatment outcomes, and (3) the slow evolution of infection requiring year-long (or even decade-long) follow-up to monitor the outcome of a given therapeutic approach [26]. Indeed, the recommended follow-up for both diseases is 10 years [10]. All these factors combine to make it virtually impossible for a single or even multicenter studies to enroll a sufficient number of well characterized, clinically homogeneous cases (for CE, in terms of cyst number, size, infected organ, localization within the organ, and stages) over a reasonably short period of time and follow-them up for long enough without significant loss to follow-up or having to change treatment strategy because of intervening situations and within the timeframe of a project funded by a funding agency. Of no less importance, such a study would probably not be ethically justified as efficacy, safety, and basic biology data all support the continuous administration regimen, which has been widely in use for the past 20 years.

European legislation also acknowledges that “In exceptional situations, studies in everyday medical practice could be requested […] where the specific scientific concern is best studied having access to data collected in everyday medical practice” [32]. All these situations apply to CE and AE. Since research to find new compounds against these parasites is progressing very slowly [11, 34], change in ABZ licensing is urgently needed to revise its administration schedule. A change in packaging would also be needed because in many countries the only presentation currently available is 1- or 3-tablet boxes that are extremely impractical for long therapies [30], adding to the very high cost of the drug and frequent “out-of-stock” situation, regularly putting patients at risk of therapy discontinuation and disease progression [35, 36].

Should new efficacy or safety data be required by the regulators to come to this decision, the way forward would be the analysis of prospectively collected data with the use of an individual patient data set, as used by Stojkovic and colleagues in their seminal study on ABZ efficacy [12]. Today, the European Register of CE (ERCE) [37] and the French and German Registries for AE [38] may provide a platform to collect prospectively clinical data from participating centers in a standardized manner, for the issue of evidence-based recommendations. However, this kind of effort requires funding and, according to European legislation, the cost of postauthorization studies should be covered by the license holders. Clearly, however, there is limited interest to invest in revising prescribing information for drugs used for neglected diseases.

We urge a coordination between expert societies, the European Medicines Agency, and the license holders to find effective and feasible ways to take action to revise the benzimidazole dosage regimens for CE and AE and ensure a fair, regular, and easy access to the appropriate treatment to those suffering from these serious diseases.
